# Interictal pontine metabolism in migraine without aura patients: A 3 Tesla proton magnetic resonance spectroscopy study

**DOI:** 10.1016/j.nicl.2021.102824

**Published:** 2021-09-20

**Authors:** Samaira Younis, Anders Hougaard, Casper E. Christensen, Mark B. Vestergaard, Olaf B. Paulson, Henrik B.W. Larsson, Messoud Ashina

**Affiliations:** a Danish Headache Center, Department of Neurology, Rigshospitalet Glostrup, Glostrup, Denmark; b Functional Imaging Unit, Department of Clinical Physiology, Nuclear Medicine and PET, Rigshospitalet Glostrup, Glostrup, Denmark; c Neurobiology Research Unit, Department of Neurology, Rigshospitalet, Copenhagen, Denmark; d Department of Clinical Medicine, University of Copenhagen, Copenhagen, Denmark

**Keywords:** CRLB, Cramér–Rao lower bounds, FWHM, full-width of half-maximum, GABA, gamma-aminobutyric ﻿acid, Glx, glutamate+glutamine, ^1^H-MRS, proton magnetic resonance spectroscopy, ^31^P-MRS, phosphorous magnetic resonance spectroscopy, ICHD, International Classification of Headache Disorders, IQR, interquartile range, total creatine, creatine + phosphocreatine, SE, standard error of the mean, SNR, signal-to-noise ratio, total NAA, *N*-acetylaspartate + *N*-acetylaspartylglutamate, Headache, ^1^H-MRS, Energy metabolism, Mitochondrial, Brainstem, Glutamate

## Abstract

In the pons, glutamatergic mechanisms are involved in regulating inhibitory descending pain modulation, serotoninergic neurotransmission as well as modulating the sensory transmission of the trigeminovascular system. Migraine involves altered pontine activation and structural changes, while biochemical, genetic and clinical evidence suggests that altered interictal pontine glutamate levels may be an important pathophysiological feature of migraine abetting to attack initiation.

Migraine without aura patients were scanned outside attacks using a proton magnetic resonance spectroscopy protocol optimized for the pons at 3 Tesla. The measurements were performed on two separate days to increase accuracy and compared to similar repeated measurements in healthy controls. We found that interictal glutamate (i.e. Glx) levels in the pons of migraine patients (n = 33) were not different from healthy controls (n = 16) (p = 0.098), while total creatine levels were markedly increased in patients (9%, p = 0.009). There was no correlation of glutamate or total creatine levels to migraine frequency, days since the last attack, usual pain intensity of attacks or disease duration.

In conclusion, migraine is not associated with altered interictal pontine glutamate levels. However, the novel finding of increased total creatine levels suggests that disequilibrium in the pontine energy metabolism could be an important feature of migraine pathophysiology.

## Highlights

• Migraine patients were scanned by a ^1^H-MRS protocol optimized to target the pons.
• Measurements were repeated on two separate days to increase accuracy.
• Pontine glutamate levels were not increased outside attacks in patients.
• Interestingly, interictal pontine total creatine levels were increased in patients.
• Imbalanced pontine energy metabolism may be a potential imaging marker in migraine.

## 1 Introduction

Sensory processing is modulated in deep brain structures by various neurotransmitters and neuropeptides (Noseda et al., 2017). One important modulator is glutamate, the major excitatory brain neurotransmitter, which also acts as a substrate in the brain energy metabolism (McKenna, 2013). In the pons, glutamate is involved in regulating the inhibitory descending pain modulation via the locus coeruleus (Hayashida et al., 2010) and the nucleus raphe magnus (Satoh et al., 1983), thus alleviating pain. Glutamate also drives activation of the dorsal raphe nucleus, regulating serotoninergic neurotransmission (Soiza-Reilly and Commons, 2011). In addition, glutamatergic mechanisms are important in regulating the sensory transmission in the trigeminovascular system (Lambert et al., 2004).

In migraine, genetic and biochemical studies have suggested abnormal regulation of the glutamate levels (Burstein et al., 2015, Hoffmann and Charles, 2018, Younis et al., 2017). Moreover, migraine medications, such as sumatriptan and topiramate, may modulate glutamatergic activity (Linde et al., 2013, Ma, 2001). Activation of the dorsal pons has consistently been reported as one of the earliest detectable events during the headache phase, suggesting that this area plays a role in generation of migraine attacks (Afridi et al., 2005a, Afridi et al., 2005b, Weiller et al., 1995, Younis et al., 2020a). In addition, migraine studies have demonstrated structural changes in the pons, ipsilateral to the pain side (Chong et al., 2017), as well as disrupted inhibitory descending regulation in the brainstem (Moulton et al., 2008). Collectively, these findings suggest that abnormal interictal glutamate levels in the pons may be an important pathophysiological feature of migraine abetting to attack initiation (Burstein et al., 2015, Hoffmann and Charles, 2018). Glutamate can be measured non-invasively in the pons by proton magnetic resonance spectroscopy (^1^H-MRS). The ^1^H-MRS protocol was optimized to explicitly target the pons (Younis et al., 2018), which is technically challenging due to the increased magnetic field inhomogeneity in that area. Using the optimized protocol, we demonstrated that glutamate levels were unchanged in the pons during attacks, compared to the interictal state (Younis et al., 2020a). Whether the levels are abnormal in the pons outside the attacks, compared to healthy controls, is unknown. We hypothesized that pontine glutamate (i.e. Glx) levels would be elevated in migraine patients outside attacks compared to healthy participants. The measurements were performed twice on separate days to increase accuracy. Further, we exploratively investigated whether pontine levels of lactate, total creatine, and total NAA differ between interictal migraine patients and healthy participants.

## 2 Material and methods

### 2.1 Participants

All participants were recruited from advertisements on a Danish website for participants to health research (www.forsogsperson.dk), hospitals or educational institutions. Episodic migraine without aura patients, diagnosed according to the International Classification of Headache Disorders (ICHD) (Headache Classification Committee of the International Headache Society (IHS), 2018), were 18–50 years of age, weighed 50–100 kg, and experienced ≥ 1 migraine attacks every other month. Exclusion criteria were shifting laterality of unilateral attacks, other primary headache disorders (except episodic tension-type headache not conflicting with episodic migraine diagnosis), no use of contraceptives, daily medication intake (except oral contraceptives), pregnant/breastfeeding females, daily smoking, history of serious somatic/psychiatric disease, drug abuse, hypo- or hypertension (systolic blood pressure > 150 mmHg or < 90 mmHg and/or diastolic blood pressure > 90 mmHg or < 50 mmHg), and MRI contraindications (including braces and teeth implants, which could cause magnetic field inhomogeneities in the pontine region). Additional exclusion criteria for healthy participants were a history of primary headache disorders (except episodic tension-type headache < 2 days per month during the past year) and first-degree family members with primary headache disorders (except episodic tension-type headache < 6 days per month) according to ICHD. All participants underwent a medical examination before each study day including a pregnancy test (female participants) before enrollment and on each study day.

The study was approved by the Ethical Committee of the Capital Region of Denmark (H-15019063) and all participants provided written informed consent in agreement with the Declaration of Helsinki of 1964 with later revisions. The study was registered at www.clinicaltrials.gov (NCT03143465) and other data of the parent study are presented elsewhere (Younis et al., 2020a, Younis et al., 2018).

### 2.2 Data acquisition

This study was part of a larger study where healthy participants underwent MRI scans on three separate study days and migraine patients underwent MRI scans on two separate study days at the same time of day within participants. Parts of the data have been published previously (Younis et al., 2020a, Younis et al., 2018). All participants were headache-free for at least 48 h, were fasting for 4 h, and were not allowed coffee, tea, alcohol, cocoa, or tobacco for 12 h before the study start.

Clinical characteristics, including pain intensity, of usual attacks of migraine patients were obtained before study days. Pain intensity was rated on a numeric rating scale ranging from 0 to 10; denoting ‘no pain’ to ‘worst imaginable pain’.

Data were acquired using a 3 Tesla Philips Achieva dStream MRI scanner (Philips Medical Systems, Best, The Netherlands) with a 32-channel phase array receiver head coil.

High-resolution anatomical images were acquired by a 3D T1-weighted turbo field echo sequence for positioning of the ^1^H-MRS-voxels. The scan sequence parameters were field of view 240 × 240 × 170 mm^3^, voxel size 1.00 × 1.08 × 1.10 mm^3^, echo time 3.7 ms, repetition time 8.0 ms and flip angle 8°. The anatomical images were segmented into gray matter, white matter and cerebrospinal fluid, using the FSL-functions BET and FAST (FMRIB Software Library, University of Oxford, Oxford, UK) and used for water-correction of metabolites concentration estimates.

**Fig. 1:**
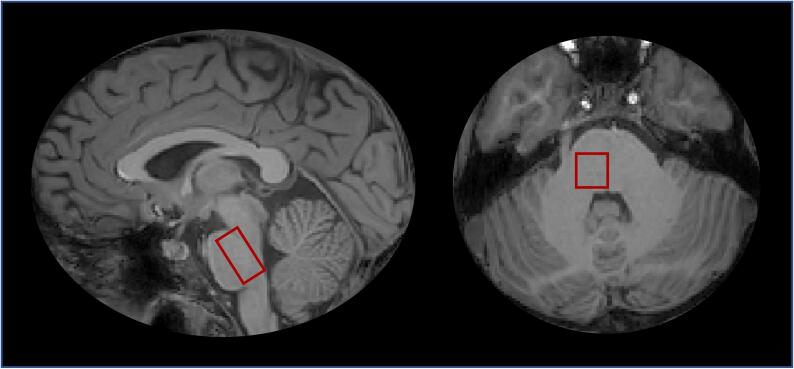
Location of the pontine ^1^H-MRS voxel.

**Fig. 2:**
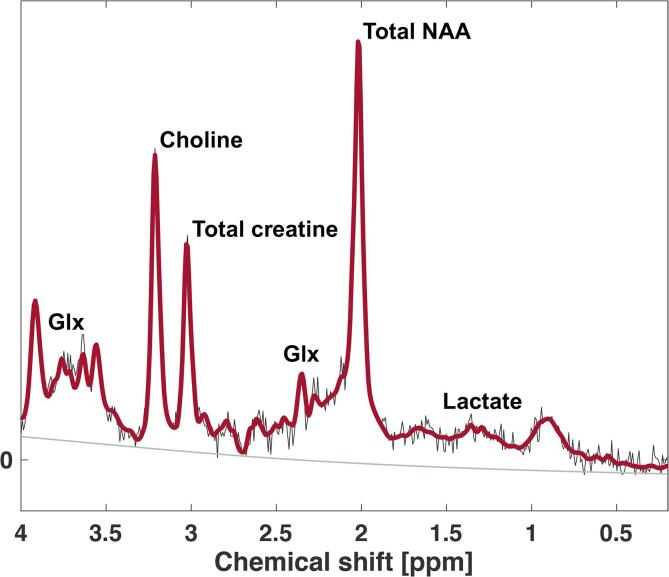
Example of representative pontine spectrum obtained from a patient. The lines represent the LCModel fit of the spectrum. The red line reflects the spectral fit and the grey horizontal line denotes the baseline. Glx: glutamate + glutamine. NAA: N-acetylaspartate. (For interpretation of the references to colour in this figure legend, the reader is referred to the web version of this article.)

Point-resolved spectroscopy pulse sequence with ^1^H-MRS voxel size of 10.5 × 12.5 × 22 mm^3^, repetition time 3000 ms, echo time 38.3 ms and 480 acquisitions with a duration of 24 min was used to measure Glx, which represents the combined concentration of glutamate and glutamine (Danbolt, 2001), lactate, total creatine (creatine + phosphocreatine) and total NAA (NAA; *N*-acetylaspartate + NAAG; *N*-acetylaspartylglutamate) (Fig. 1). Imaging protocol was optimized to target small deep brain areas such as the pons and reducing potential partial volume effects by using small voxel size and increased acquisitions number. The unsuppressed water signal was obtained from the voxel and used as internal reference for quantifying the absolute concentration of metabolites (Christiansen et al., 1993). The water concentration in each acquired spectroscopy voxel was estimated based on the grey matter, white matter and cerebrospinal fluid segmentation of voxels using the structural imaging (Quadrelli et al., 2016). Post-processing and metabolite quantification were performed using LCModel (Version 6.3-1F, Toronto, Canada). Concentration estimates of the metabolites were corrected for water contribution to the pontine measurement. The spectral quality parameters, signal-to-noise ratio (SNR), linewidth (full-width of half-maximum; FWHM) and Cramér–Rao lower bounds (CRLB), were extracted from LCModel. Blinded visual inspection of LCModel spectra was performed to exclude spectra with poor spectral fit, gross outliers, due to e.g. motion, and artefacts. Further spectral exclusion criteria were FWHM > 0.1 ppm and SNR < 10. An example of a pontine spectrum from a patient is presented in Fig. 2.

### 2.3 Statistical analysis

Results are reported as mean and standard error of the mean, unless stated otherwise. Primary outcome was difference in levels of Glx, lactate, total creatine and total NAA in the pons between patients (measured on two separate study days) and healthy participants (measured on three separate study days). A linear mixed model (1) was used for each metabolite with grouping (migraine patients and healthy participants) as fixed effect and covariates of age and sex to correct for possible age and sex differences between cohorts. Participant identification is the random effect (μ) and ε denotes the random error.

(1) $$Y_{\mathrm{value}}=\beta 0\;+\beta 1\;x\;group\;+\beta 2\;x\;age\;*\;sex+\beta 3\;x\;group*age*sex+\mu +\varepsilon$$

As an explorative analysis, we investigated the correlation between days since the last migraine attack (counting from the first study day), monthly attack frequency, usual pain intensity of attacks and disease duration to the metabolite levels for the first study day using a linear regression model. We were unable to conduct a priori sample size calculations for inclusion of participants as the data of the current study are part of a larger parent study. Median and interquartile ranges (IQR) were reported for difference in Glx and total creatine levels in the morning (n = 13) and afternoon (n = 3) for healthy participants, due to sample sizes.

In supplementary explorative analysis, differences in the spectral quality parameters (i.e. SNR, FWHM and CRLB’s of metabolites) between patients and healthy participants were investigated. A linear mixed model was used for each spectral quality parameter with grouping (migraine patients and healthy participants) as fixed effect and participant identification as the random effect.

All statistical analyses were performed in R (Version 3.5.1). P values were reported as two-tailed with a level of significance of 5%.

## 3 Results

### 3.1 Participants and clinical data

**Fig. 3:**
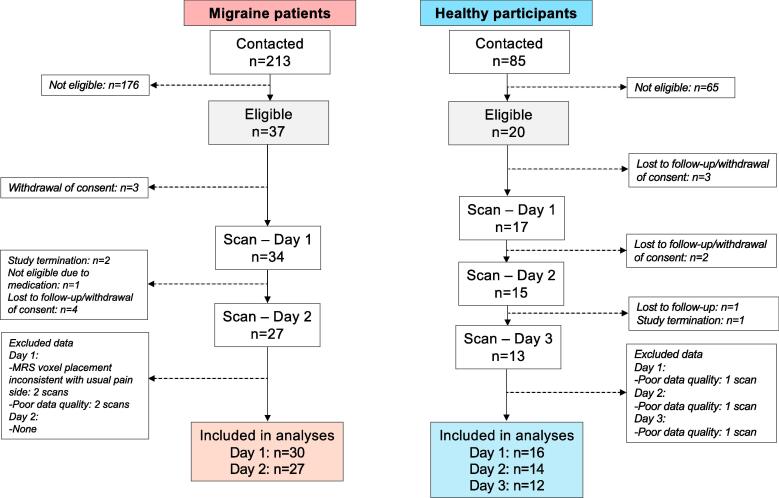
Inclusion process. Two migraine patients did not continue to study day 2 as the study was finalized. One patient initiated daily medication intake after completion of study day 1, which was an exclusion criterion, and did not continue to study day 2. Data were excluded from two different migraine patients due to poor spectral quality, whereof one patient did not complete the second study day. Data were excluded from the same healthy participant due to poor spectral quality.

Data from 33 migraine patients (29 females, p = 0.025; mean age 25.4 ± 5.1 years, median 24.5, range 20–47, p = 0.058) and 16 healthy participants (9 females; mean age 22.9 ± 3.5 years, median 22.5, range 18–30) were included in the final analysis (Fig. 3). Migraine patients had a median frequency of 1.25 (range 0.5–10) migraine days per month (n = 33). Usual pain intensity was 8.2 ± 1.1 (missing data from three patients) as rated on the 0–10 scale. Median days since the last attack to the first study day were 16 days (IQR 10.5–39). The median days between study day 1 and 2 in migraine patients were 14 days (IQR 7.25–25.75). In healthy participants, the median days between study day 1 and 2 were 10.5 days (IQR 7–14) and 8 days (IQR 7–14) between study days 2 and 3.

MRI scans were carried out in the morning for all patients (except for one in the afternoon) and in the afternoon for healthy participants (except for three participants in the morning) due to feasibility.

There were no differences in spectral quality parameters (i.e. SNR, FWHM and CRLBs) between patients and healthy participants (Supplementary Table 1).

### 3.2 Glx

We found no difference in the pontine levels of Glx between migraine patients and healthy participants (Table 1). There was no correlation between days since the last migraine attack and the Glx levels. Migraine frequency per month, pain intensity of usual migraine attacks or duration of disease did not correlate to the Glx levels either.

**Table 1:** Glx, lactate, total NAA and total creatine concentration estimates in patients and healthy participants.

	Migraine patients mean mmol/L ± SE	Healthy participants mean mmol/L ± SE	Effect estimate and 95% CI	P
Glx	8.92 ± 0.28	8.45 ± 0.69	0.48, −0.05–1.00	0.098
Total creatine	3.95 ± 0.12	3.62 ± 0.29	0.33, 0.10–0.56	0.009
Total NAA	9.91 ± 0.21	9.94 ± 0.51	−0.03, −0.42–0.37	0.906
Lactate	0.79 ± 0.13	0.76 ± 0.31	0.03, −0.21–0.26	0.824

Exclusion of one extreme Glx outlier of migraine data (∼4 standard deviations from mean) did not change results (p = 0.071). Absolute mean and standard error of the mean (SE) reported from mixed model. CI: confidence interval. Glx: glutamate + glutamine. NAA: *N*-acetylaspartate.

Due to previous studies reporting diurnal fluctuations in the brain Glx levels (Volk et al., 2018), we further considered the Glx levels of healthy participants scanned in the morning (median 8.0, IQR 7.71–8.29, n = 3), and in the afternoon (median 7.89, IQR 7.35–8.27, n = 13), which indicate no difference.

In the 24 migraine patients, from which data of both study days were included, there was no difference in the Glx levels between the two days (p = 0.472). In healthy participants, there were no differences in the Glx levels between the three study days (p > 0.100).

### 3.3 Other metabolites

Total creatine level was 9% higher in migraine patients compared to healthy participants (p = 0.009). Median total creatine levels in the morning (median 4.20, IQR 3.97–4.49, n = 3) were not different from levels in the afternoon (median 4.34, IQR 4.04–4.58, n = 13). There was no difference in the pontine levels of total NAA or lactate between migraine patients and healthy participants (Table 1).

We found no correlation between days since the last migraine attack and the level of total creatine, total NAA or lactate. Migraine frequency per month, pain intensity of usual attacks and duration of disease were neither correlated to the total creatine, total NAA or lactate levels.

In the 24 patients, with completed data from both study days, the total NAA level was higher on the first study day, compared to the second day (p = 0.043). However, when excluding one extreme outlier (second day,∼ 3.5 SD from mean), the difference was no longer statistically significant (p = 0.083). There were no differences in the lactate (p = 0.394) or total creatine (p = 0.104) levels between the two days. In healthy participants, there was no difference in the total creatine, total NAA or lactate levels between the three study days (p > 0.200).

## 4 Discussion

Proton magnetic resonance spectroscopy (^1^H-MRS) in migraine with repeated measurements revealed no alterations in the pontine Glx levels compared to controls. Interestingly, we found markedly increased levels of total creatine in the pons of migraine patients. Neither Glx nor total creatine correlated to migraine frequency, days since the last attack, usual pain intensity of attacks or disease duration.

### 4.1 Glutamate

Elevated glutamate levels, as measured by ^1^H-MRS, have been coupled to increased neuronal activation (Bednařík et al., 2015, Betina Ip et al., 2017, Cleve et al., 2017, Stanley and Raz, 2018).

In the current study, we investigated Glx, which represents the combined concentration estimate of glutamate and glutamine, as the signals of these two metabolites are inseparable at 3 Tesla (Deelchand et al., 2014, Younis et al., 2020b). However, majority of the combined concentration (i.e. glutamate + glutamine) in the brain consists of glutamate (∼80%) in healthy volunteers (Tkáč et al., 2009), making Glx a feasible parameter to assess glutamate levels at 3 Tesla. The present data do not show altered Glx levels, as measured by ^1^H-MRS, in the pons of migraine patients in the interictal state. Our recent study also showed no alterations in the pontine Glx levels during attacks compared to attack free days (Younis et al., 2020a). Other brain regions may modify the interictal neuronal threshold in migraine via glutamate-dependent mechanisms. The thalamus relays, projects and modulates sensory information to multiple brain regions under the influence of neurotransmitters and neuropeptides (Noseda et al., 2017, Younis et al., 2019). Increased glutamate levels in the thalamus were reported outside of attacks (Bathel et al., 2018). Furthermore, studies of migraine without aura reported interictally increased glutamate levels in the anterior paracingulate (included migraine with aura data) and visual cortices, which receive projections from the thalamus (Bathel et al., 2018, González de la Aleja et al., 2013, Younis et al., 2017, Zielman et al., 2017). However, some studies also reported normal interictal glutamate levels in the insula, anterior cingulate, posterior cingulate, and visual cortices in migraine with and without aura, as well as normal ictal levels in the visual cortex of migraine with aura (Aguila et al., 2015, Arngrim et al., 2016, Becerra et al., 2016, Fayed et al., 2014, Prescot et al., 2009)

Another question is whether the glutamate levels are altered at a different stage of the migraine phase. Repeated measurements during the course of a migraine attack may elucidate if alterations in the glutamate levels appear closer to an impending attack, and/or in the early stages of an attack. Future pontine investigation in migraine may be expanded by adding measurement of the major inhibitory neurotransmitter, gamma-aminobutyric acid (GABA), possibly at increased magnetic field strengths (Younis et al., 2020b).

In the present study, the majority of participants were females in both groups. While we corrected for possible sex differences in our statistical analyses, we cannot exclude sex potentially influencing the metabolite levels. One common methodological limitation of MRS is that the intra- and extracellular pools of glutamate are inseparable with the currently available techniques. However, increased glutamate levels, detected by ^1^H-MRS, likely reflects the release of glutamate into the extracellular space due to neuronal excitability (Bednařík et al., 2015, Betina Ip et al., 2017, Cleve et al., 2017, Foerster et al., 2013, Stanley and Raz, 2018). Another common limitation of 3 Tesla ^1^H-MRS is the minor contribution of glutamine to the Glx signal (Tkáč et al., 2009). Measurement at higher magnetic field strengths, such as 7 Tesla, allows reliable separation of the glutamate and glutamine signal in the pons (Younis et al., 2020b). The ^1^H-MRS voxel was larger than the specific nuclei involved in the glutamatergic migraine mechanisms. The size of the voxel was necessary to acquire sufficient SNR. Nevertheless, we assumed that abnormally increased glutamate levels, involving the specific nuclei, would be reflected in the acquired voxel. A longer echo time would be optimal for detection of lactate; however, this would compromise detection of Glx, which requires a short echo time.

One previous study of healthy participants demonstrated a detectable change of at least ∼ 6% of glutamate in pons with an estimated variability of 6.9% after pharmacological intervention (Younis et al., 2018). Based on these variables, a post hoc calculation (significance level: 5%, power: 80%) showed that a sample size of 22 participants in each group would be sufficient in the current study. We included data from 33 patients and 16 healthy participants. This setup merely yields a 2.5% increase of the detection threshold compared to the sample size of 22 patients in each group. Thus, it is unlikely that data from additional six healthy participants would markedly change the results. Another major strength in the current study is the application of a ^1^H-MRS protocol designed to target the small deep brain region of the pons, which is technically challenging. Furthermore, accuracy was increased as we applied within-subject repeated measurements. This accounts for temporal changes, in contrast to previous ^1^H-MRS studies in migraine.

### 4.2 Total creatine

Total creatine level was increased in migraine patients during their interictal state. In a previous ^1^H-MRS study, we found increased total creatine levels in the pons during attacks, suggesting involvement of abnormal energy metabolism in migraine (Younis et al., 2020a). Other studies reported normal interictal and ictal total creatine levels in the visual cortex compared to healthy controls (Arngrim et al., 2016, Reyngoudt et al., 2010a, Sarchielli et al., 2005, Younis et al., 2017). Total creatine is a component of the energy metabolism and constituted by the combined concentration estimates of creatine and phosphocreatine. Creatine kinase converts phosphocreatine to creatine while releasing energy in the form of ATP (Wyss and Kaddurah-Daouk, 2000). The cascade is reversed via the mitochondrial oxidative phosphorylation (Wyss and Kaddurah-Daouk, 2000). The phosphocreatine pool is an energy buffer and rapidly shuttles ATP to subcellar sites in need of energy. Thus, the phosphocreatine-creatine kinase system plays a role in regulating the ATP levels according to demand. Additionally, creatine is transported into the cells to regulate the total creatine levels necessary for the energy buffer system (Sartorius et al., 2008, Wyss and Kaddurah-Daouk, 2000). The estimates of creatine and phosphocreatine are conjugated with the currently available ^1^H-MRS techniques at clinical magnetic field strengths (e.g. 3 Tesla). This challenges interpretation as we are unable to conclude in which direction the phosphocreatine-creatine kinase system may be affected. Investigations by phosphorous (^31^P) MRS allow for separation of the components of the energy metabolism (Chen et al., 2018, Chen et al., 1997).

Previous ^31^P-MRS studies in migraine have reported decreased phosphocreatine levels during and outside attacks in the cortex (i.e. frontal, temporo-parietal and occipital), indicating that the creatine kinase system may be affected (Younis et al., 2017). One study even suggested decreased ATP levels along with decreased phosphocreatine levels in the visual cortex (Reyngoudt et al., 2010b). Moreover, these studies reported decreased phosphorylation potential indicating insufficient available energy (Younis et al., 2017). Collectively, previous findings, albeit detected in more cortical structures, suggest disequilibrium in the energy metabolism (ATP production vs consumption), where the supply of (phospho-)creatine is insufficient to meet the increased ATP synthesis rate (Younis et al., 2017). In support, a possible component of mitochondrial dysfunction in migraine was suggested in a genetic study (Guo et al., 2016). Future studies, applying refined neuroimaging techniques, are necessary to clarify the observed interictal pontine total creatine level in the current study. The possible association between altered (phospho-)creatine concentration and energy metabolism could potentially be a biomarker of migraine.

## 5 Conclusion

We found no alterations in the pontine glutamate levels in migraine patients without aura using repeated measurements to increase accuracy. While the exact implication of increased total creatine levels in the pons needs to be examined in future studies, we suggest that disequilibrium in the pontine energy metabolism could be a potential imaging marker in migraine.

## 6 Fundings

This work was supported by the Research Foundation of Rigshospitalet (E-23327-02) and Lundbeck Foundation (R155-2014-171). Funding sources had no influence on study design, patient inclusion or data interpretation.

## 7 Ethics approval and consent to participate

The study was approved by the Ethical Committee of the Capital Region of Denmark (H-15019063) and conducted according to the regulations of the Danish Data Protection Agency. All participants provided written informed consent in agreement with the Declaration of Helsinki of 1964 with later revisions. ﻿

## 8 Consent for publication

Not applicable.

## CRediT authorship contribution statement

**Samaira Younis:** Conceptualization, Methodology, Investigation, Formal analysis, Visualization, Writing – original draft. **Anders Hougaard:** Conceptualization, Methodology, Writing - review & editing. **Casper E. Christensen:** Investigation, Writing - review & editing. **Mark B. Vestergaard:** Methodology, Formal analysis, Writing - review & editing. **Olaf B. Paulson:** Conceptualization, Writing - review & editing. **Henrik B.W. Larsson:** Conceptualization, Methodology, Writing - review & editing. **Messoud Ashina:** Supervision, Project administration, Conceptualization, Writing - review & editing, Supervision.

## Declaration of Competing Interest

The authors declare the following financial interests/personal relationships which may be considered as potential competing interests: MA is a consultant, speaker or scientific advisor for AbbVie, Allergan, Amgen, Alder, Biohaven, Eli Lilly, Lundbeck, Novartis, and Teva, and primary investigator for Alder, Amgen, Allergan, Eli Lilly, Lundbeck, Novartis and Teva trials. MA has no ownership interest and does not own stocks of any pharmaceutical company. MA serves as associate editor of *Cephalalgia*, associate editor of the *Journal of Headache and Pain*. MA is president of the International Headache Society. CEC has received personal fees for lecturing from Teva and serves as consultant for Teva. The remaining authors report no competing interests.
