# Supplementary material for: Interictal pontine metabolism in migraine without aura patients: A 3 Tesla proton magnetic resonance spectroscopy study
Source: Neuroimage Clin. 2021 Sep 20;32:102824. doi: 10.1016/j.nicl.2021.102824 (PMC8498457; doi:10.1016/j.nicl.2021.102824)
Supplement: Supplementary data 1 [file mmc1.docx]

**Supplementary Table 1.** Spectral quality parameters

|  | Migraine patients  mean ± SE | Healthy participants  mean ± SE | P |
| --- | --- | --- | --- |
| SNR | 16.39 ± 0.68 | 17.54 ± 0.55 | 0.099 |
| FWHM | 0.05 ± 0.00 | 0.05 ± 0.00 | 0.928 |
| Glx – CRLB | 9.87 ± 0.33 | 9.30 ± 0.25 | 0.089 |
| Total creatine – CRLB | 4.32 ± 0.19 | 4.24 ± 0.15 | 0.683 |
| Total NAA – CRLB | 3.49 ± 0.17 | 3.26 ± 0.13 | 0.176 |
| Lactate – CRLB | 84.47 ± 16.62 | 85.68 ± 13.16 | 0.943 |

Exclusion of one extreme lactate outlier of healthy data (~ 4.7 standard deviations from mean) did not change outcome (p=0.236). Removing corresponding lactate value did not change output either for group differences reported in Table 1 (p= 0.652). Absolute mean and standard error of the mean (SE) reported from mixed model. CRLB: Cramér–Rao lower bounds. SNR: signal noise ratio. FWHM: full-width of half-maximum, ppm. Glx: glutamate+glutamine. NAA: N-acetylaspartate.
